# Galectins as pivotal components in oncogenesis and immune exclusion in human malignancies

**DOI:** 10.3389/fimmu.2023.1145268

**Published:** 2023-02-03

**Authors:** Nikiforos-Ioannis Kapetanakis, Pierre Busson

**Affiliations:** ^1^ Research & Development (R&D), 4D Lifetec, Cham, Switzerland; ^2^ Host-Tumor Interactions in Head and Neck Carcinoma: Exploration and Therapeutic Modulations, Centre National de la Recherche Scientifique (CNRS), Unité Mixte de Recherche(UMR) 9018 - METabolic and SYstemic aspects of oncogenesis for new therapeutic approaches (METSY), Gustave Roussy and Université Paris-Saclay, Villejuif, France

**Keywords:** galectins, cancer, immune evasion, tumor microenvironment, galectin inhibitors, combination immunotherapy

## Abstract

Galectins are galactoside-binding proteins, exerting numerous functions inside and outside the cell, particularly conferring adaptation to stress factors. For most of them, aberrant expression profiles have been reported in the context of cancer. Albeit not being oncogenic drivers, galectins can be harnessed to exacerbate the malignant phenotype. Their impact on disease establishment and progression is not limited to making cancer cells resistant to apoptosis, but is prominent in the context of the tumor microenvironment, where it fosters angiogenesis, immune escape and exclusion. This review focuses mainly on Gal-1, Gal-3 and Gal-9 for which the involvement in cancer biology is best known. It presents the types of galectin dysregulations, attempts to explain the mechanisms behind them and analyzes the different ways in which they favor tumour growth. In an era where tumour resistance to immunotherapy appears as a major challenge, we highlight the crucial immunosuppressive roles of galectins and the potential therapeutic benefits of combinatorial approaches including galectin inhibition.

## Introduction

Galectins are animal lectins, which specifically bind glycans containing disaccharides with β-galactoside bonds, such as N-acetyllactosamine. These glycans are carried either by proteins, known as glycoproteins, with N- or O-linked glycosylation, or by lipids, known as glycolipids (1). Encoded by the *LGALS* genes, 16 galectins have been identified in mammals and 12 in humans (2). One could argue that galectins have two faces. On the one hand, their family has conserved a remarkable homogeneity. Bearing highly conserved regions (3), they are characterized by structural overlap, collective inhibition by the same substrate (lactose or N-acetyllactosamine) or chemical modification (glycan sialylation) and target homogeneity, strongly interacting with beta-1-3 or 1-4 galactoside di-saccharide motifs (1). On the other hand, works prove a remarkable evolutionary diversification in terms of cellular and tissular functions (3, 4).

**Figure 1:**
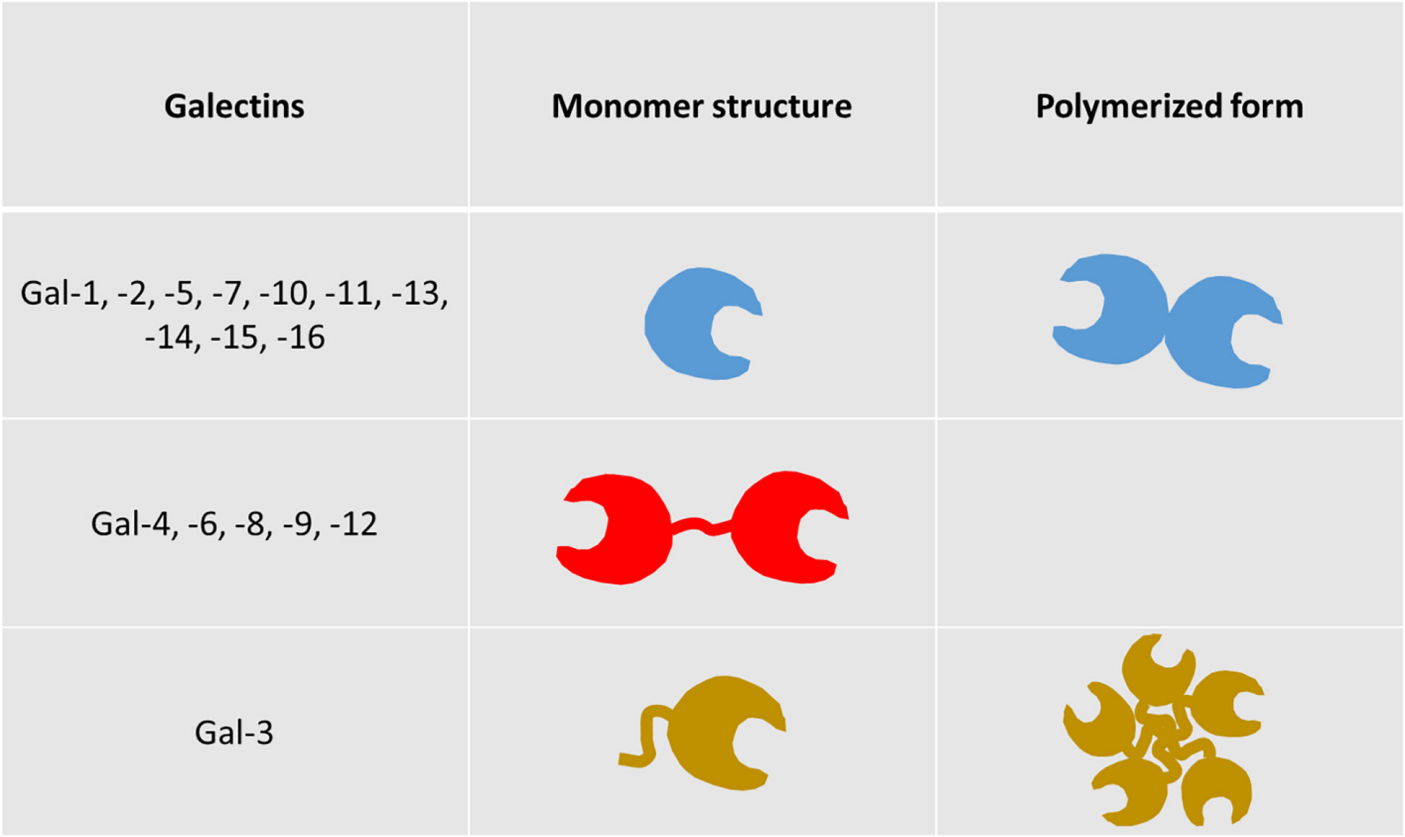
Structural types of galectins. Galectins are categorized in three distinct groups, based on their structure. The first group contains members bearing a single carbohydrate recognition domain (CRD), while the second comprises of members with two CRDs, connected by a short peptide sequence (tandem repeat-type galectins). Galectin-3 is the sole member of the third group, as it combines a single CRD with a unique amino-terminal non-lectin domain (chimera-type galectin). Members of groups 1 and 3, bearing a single CRD, are able to polymerize, exhibiting new and distinct functional features compared to their monomeric forms (5).

One basic common feature of all galectins is the existence of at least one highly homologous carbohydrate recognition domain (CRD). They are divided into 3 groups. The first group bears a single CRD and forms monomers or homodimers (Gal-1, -2, -5, -7, -10, -11, -13, -14, -15, -16), while the second possesses two CRDs linked by a short peptide (Gal-4, -6, -8, -9, -12). Gal-3 constitutes the third group on its own, combining a CRD and a large amino-terminal part, making it able to pentamerize (**Figure 1**). Unlike the majority of lectins, they are not only membrane-associated, but also exhibit intra- and extracellular presence (6). They are found in the cytosol or associated with the endomembrane system, in the nucleus, at the surface of the plasma membrane, in the extracellular matrix, or in the extracellular fluid with possible diffusion in the blood. Analyzing galectin binding on the surface of cells with targeted stable glycosylation mutations, Nielsen and colleagues showed that Gal-1, -3, -8N, -9N and -9C bind the cells with high affinity, while Gal-2, -4C, -4N, -7, and -8C demonstrated insignificant binding. Furthermore, galectins exhibited high affinity only for N-glycans, with the exception of Gal-8N, which binds both types of glycans with high affinity and, secondarily Gal-1 and Gal-9N (1). An older analysis suggests that Gal-3 binds roughly 50% of all glycoproteins in serum, whereas Gal-1 and, even less, galectins -8 and -9 bind substantially smaller fractions. Other galectins (Gal-2, -4, and -7) bind only trace amounts of serum glycoproteins (7).

Although the body of literature is inconclusive, sometimes even self-contradicting regarding the impact of the different galectins in cancer, it is now becoming clear that Gal-1, Gal-3 and Gal-9, are consistently involved in the various hallmarks of oncogenesis in a pro-tumorigenic way. As the volume of publications in this topic is impossible to fit in a single publication, our initial purpose is to present the different ways through which dysregulated galectins may propel tumor progression. Gradually focusing on the three aforementioned members, this review emphasizes on their contribution in the formation of a robustly immunosuppressive tumour microenvironment. Finally, we provide the rationale for targeting galectins in cancer management and we update the scientific community about ongoing pre-clinical and clinical efforts. We are confident that this work can improve our understanding of galectin involvement in disease progression and better highlight the potential of their inhibition to enhance the clinical efficacy of novel and conventional therapeutic approaches.

## Overview of galectin dysregulations in human malignancies

So far, 7 galectins have been reported to be dysregulated in a variety of cancers, associated with various aspects of oncogenesis, disease progression and metastasis. Galectin dysregulations do not always have the form of an upregulated expression in cancer cells. Changes in their intracellular localization have also been linked with tumour aggressiveness, prognosis and response to therapy. Furthermore, increased galectin presence at the cell surface and/or increased extracellular secretion are frequent characteristics of various tumours. Of note, increased galectin detection inside the tumour has been often found to originate from non-malignant cells like fibroblasts in the tumour stroma, or tumour-associated macrophages. Gal-1, Gal-3 and Gal-9 are the most abundant galectins and the ones to have been linked with cancer the most (8, 9). However, works have also linked Gal-2, Gal-4, Gal-7 and Gal-8 with different aspects of disease progression. **Table 1** provides substantial, if not exhaustive, information about the various types of galectin dysregulations reported in the spectrum of human malignancies.

**Table 1:** Classification of dysregulated galectin phenotypes in cancer, according to cancer type, galectin member, type of dysregulation and impacted compartment of the malignant cell or the tumour microenvironment.

Type of cancer	Increased expression in malignant cells	Increased nuclear localization in malignant cells	Increased cytoplasmic concentration in malignant cells	Increased expression in stromal cells	Increased extracellular release
Colon	Galectin-3 (10)		Galectin-3 (11)	Galectin-1 (12)	Galectin-2 (13)
					Galectin-3 (14)
					Galectin-4 (13)
					Galectin-8 (13)
Breast	Galectin-7 (15)				Galectin-2 (13)
	Galectin-9 (16)				Galectin-3 (17)
					Galectin-4 (13)
					Galectin-8 (13)
NSCLC	Galectin-3 (18)				
Prostate			Galectin-3 (19)	Galectin-1 (20)	
Head and neck	Galectin-3 (18)			Galectin-1 (20)	Galectin-9 (21)
	Galectin-9 (21)				
Glioma	Galectin-1 (22)			Galectin-9 (23)	
	Galectin-9 (24)				
Ovarian	Galectin-1 (25)	Galectin-7 (15)	Galectin-1 (25)	Galectin-1 (26)	
	Galectin-9 (27)		Galectin-7 (25)		
Endometrial	Galectin-1 (18)				
Pancreas	Galectin-3 (18)			Galectin-1 (28)	Galectin-9 (29)
	Galectin-9 (29)			Galectin-9 (29)	
Stomach	Galectin-3 (18)				
Kidney	Galectin-3 (18)			Galectin-9 (30)	
	Galectin-9 (30)				
Bladder	Galectin-1 (31)				
	Galectin-3 (31)				
Thyroid	Galectin-1 (32)				
	Galectin-3 (32)				
Hepatocellular	Galectin-1 (33)				
	Galectin-3 (18)				
Melanoma	Galectin-9 (34)				Galectin-9 (35)
Chronic Lymphoid Leukemia	Galectin-9 (36)				Galectin-9 (36)
Acute myeloid Leukemia	Galectin-9 (37)				Galectin-9 (37)
Acute lymphoid leukemia	Galectin-1 (38)				

Compared to surrounding normal tissue, malignant cells exhibit increased Gal-1 expression in bladder, endometrial and thyroid cancer, cholangiocarcinoma and gliomas, correlating with disease grade in the first (18). Although Gal-1 is not abundant in malignant cells of colon and head and neck squamous cell carcinomas (HNSCC), it is abundant in their stromal cells. This was also observed in cancer cell-associated stroma invaded by pancreatic cancer cells. In prostate cancer, expression of Gal-1 in the tumour stroma was found to be an independent predictor of tumour progression, confirmed by prostate-specific antigen (PSA) increase (20). Conditioned medium from prostate cancer cells increased the detection of Gal-1 in human umbilical vein endothelial cells (HUVECs), as well as the cell-to-cell adhesion of the cancer cells to a HUVEC monolayer. This also resulted in secretion of Gal-1 by stromal fibroblasts. These findings suggest that Gal-1 in the tumour stroma can be induced by soluble factors secreted by cancer cells. One of these factors might be extracellular Gal-3 (39). Finally, Schulz et al. reported that increased expression of Gal-1 by either cancer or stroma cells is an indicator of poor prognosis in ovarian cancer (25).

Increased Gal-3 in cancer cells has been reported in thyroid and central nervous system malignancies, head and neck squamous cell carcinoma (HNSCC), pancreas, bladder, stomach and renal carcinomas. In colon and astrocytic tumours which have variable expression of Gal-3, its abundance correlates with a pejorative outcome (18). Interestingly, the balance of Gal-3 localization between the nucleus and the cytosol also seems to be a prognostic indicator, as a shift towards the second is associated with worse prognosis in breast cancer, while its nuclear localization is mainly a positive prognosticator, as shown for ovarian cancer (25). In addition to altered expression inside the malignant cells, tumour-associated macrophages are also a major intratumoral source of Gal-3 (40). Gal-1 and Gal-3 correlate with immune evasion and disease progression. Sharing many functional features, they are reported to be upregulated in increasingly hypoxic environments, possibly contributing to the enhanced aggressiveness and invasiveness of tumour cells. Intriguingly, Gal-1 is increased in pancreatic cancer cells treated with Gal-3 inhibitors, compensating the reduction of Gal-3 (41).

Galectin-9 constitutes a particular case, as the compartment where it is upregulated determines whether it is a positive or a negative prognostic factor. In chronic lymphocytic leukemia (CLL), the most common adult leukemia, it was found to be significantly increased in the serum of patients versus control subjects, being proportionally associated with treatment failure (36). On the other hand, Okoye and colleagues recently studied Gal-9 expression in peripheral and tumour-infiltrating CD4^+^ and CD8^+^ T cells in virus-associated solid tumours and discovered that its expression defines dysfunctional T cells with impaired effector capacities, indicating poor response to anti-PD-L1 immunotherapy (42). What is intriguing for Gal-9 however, is that its expression pattern inside malignant cells illustrates an opposite phenotype. A meta-analysis of Gal-9 expression in solid tumours revealed that high Gal-9 expression in malignant cells clearly correlates with a better cancer-specific survival of the patients (43). Another work in non-small cell lung cancer (NSCLC) is in line with these findings, showing that patients with low Gal-9 levels on tumour cells or high Gal-9 on tumour-infiltrating lymphocytes (TILs), as seen with immunohistochemistry, were more likely to have poor prognosis (44).

Galectin-7 is expressed in abnormally high levels most notably in breast and ovarian cancer cells. In ovarian cancer, high Gal-7 has been linked with poor prognosis and increased residual disease post-surgery, while it is mostly located in the nucleus (15). More recently, it was proven that intracellular levels of Gal-7 are also upregulated in a positive self-amplification pathway, following transcriptional activation after re-entry of extracellular Gal-7 (45).

Less is known regarding Gal-4, although its impact on cell adhesion is thought to negatively regulate metastasis. In pancreatic cancer cell lines, Gal-4 expression proved to be inversely correlated with the migratory properties of the cells (46). Higher Gal-8 expression in the nucleus of different types of breast cancer cells is consistently linked with favorable prognosis and improved overall survival (47). On the contrary, similarly to Gal-9, serum levels of Gal-2, Gal-4 and Gal-8 are greatly increased in colon and breast cancer patients and seem to be involved in metastasis (13). Taken together, these findings create a consensus, distinguishing Gal-1 and Gal-3 from the other galectins, as these are the only members of the galectin family seen to have strong tumour-promoting properties both inside and outside the malignant cells.

## Causative factors of galectin dysregulations in the context of human malignancies

The mechanisms of these dysregulations are quite diverse and warrant further investigation. Their exhaustive presentation is outside the scope of this work, but we intend to highlight here what seem for us to be striking examples.

### Interplay between galectins and *TP53*-associated pathways

**Figure 2:**
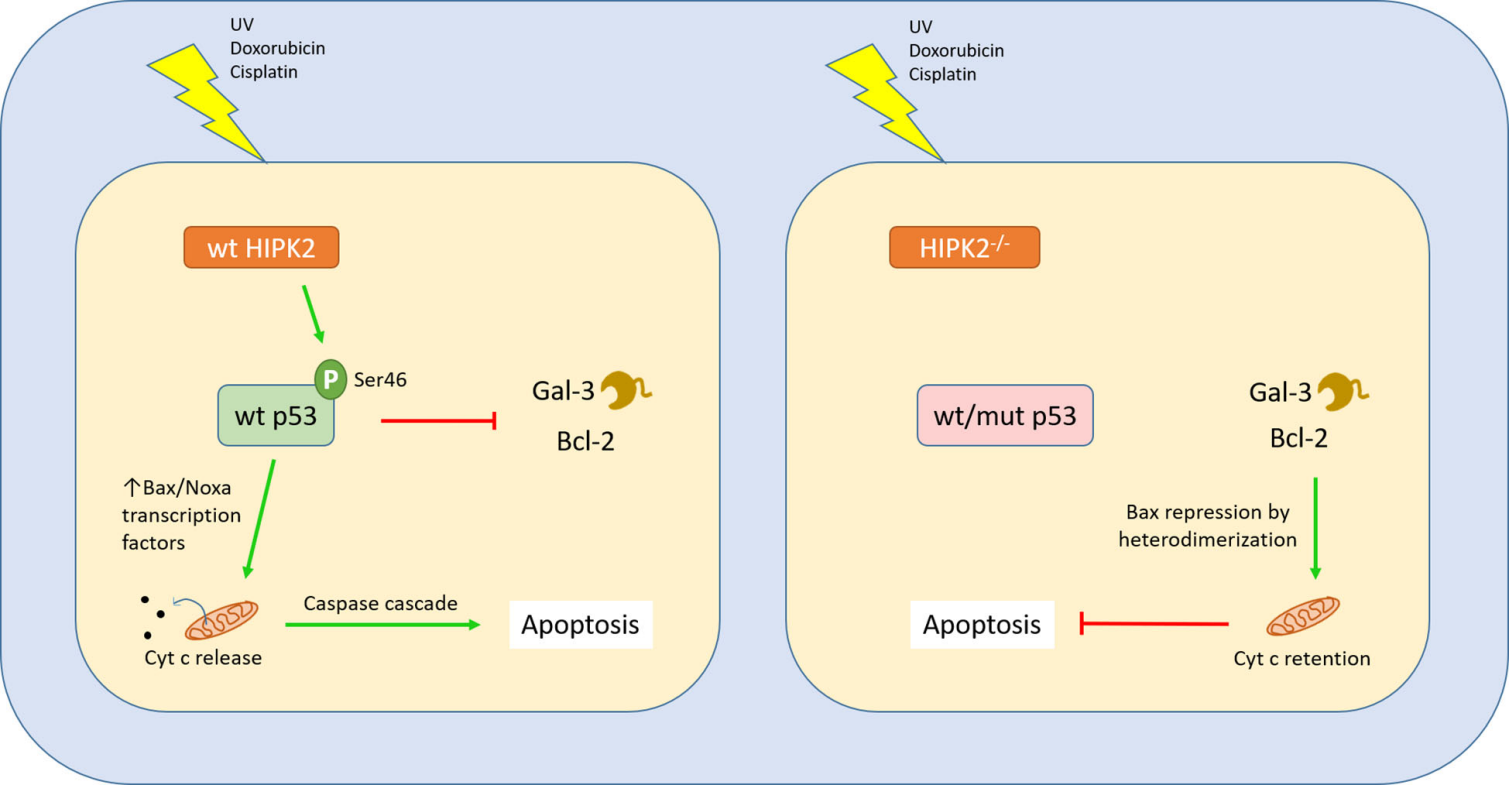
Interplay between Gal-3 and the p53 apoptotic pathway. The p53 is a master regulator of cell cycle and apoptosis, induced by cellular exposure to a variety of genotoxic damage agents or stress signals. In order to initiate apoptosis, p53 switches to its activated form through phosphorylation at serine 46 by the Ser/Thr kinase HIPK2. This modification confers affinity for promoters of genes involved in the apoptotic cascade. The downstream steps have been well characterized and can be found in other works, leading to mitochondrial disintegration and release of cytochrome C, eventually triggering apoptosis through activation of the caspase pathway. When wild type p53 is activated by Ser46 phosphorylation, it binds to the promoter of Gal-3, negatively regulating its transcription. On the other hand, when HIPK2 is absent or *TP53* bears mutations leading to a dysfunctional p53, Gal-3 is upregulated in response to stress signals (53). Gal-3 acts similarly to the apoptosis-inhibiting family of Bcl2 proteins, through a conserved peptide motif, preserving mitochondrial integrity and conferring cellular resistance to apoptotic cues (55).

AML cells with wild-type p53 exhibited enhanced apoptosis following Gal-3 inhibition (48). A similar connection between wt p53 and Gal-1 has also been described in gliomas (49). Another work shows that, in p53^-/-^ pancreatic ductal adenocarcinoma, Gal-1 is upregulated and confers enhanced tumour aggressiveness and shorter survival of the mice (50). Campion and colleagues proved that transfection of breast cancer cell lines with vectors encoding mutant p53 resulted in the upregulation of another galectin with tumour-promoting properties, Gal-7. Doxorubicin treatment of breast cancer cells harboring mutant p53 also produced the same result, *via* NF-κB activation, but this did not occur when the mutant p53 was knocked down (51). Finally, Gal-7 was found by the same group to accelerate degradation of wt p53 in a carbohydrate-binding-independent manner (52). Cecchinelli and colleagues demonstrated that the homeodomain-interacting protein kinase 2 (HIPK2), a serine-threonine kinase responsible for the activation of p53 *via* phosphorylation, negatively regulates Gal-3. HIPK2-mediated repression of galectin-3 is an important step following the induction of the p53-dependent apoptosis pathway, while forced expression of Gal-3 prevents it (53). Lavra and colleagues further showed that impaired HIPK2 in thyroid carcinomas results in a strong presence of Gal-3, despite a wild type p53 (54). Taken together, these findings suggest that mutated or dysfunctional p53, or defects in factors upstream in the p53-mediated apoptosis pathway may trigger galectin upregulation with a cell-protecting role, upon stress signals (**Figure 2**).

### Tumour hypoxia

Numerous works indicate the upregulation of galectins in response to hypoxia and nutrient deprivation, in a mechanism regulated by HIF-1α and HIF-2 (56–61). Hypoxia is known to be a fundamental force behind tumour aggressiveness and resistance to various therapeutic modalities, including checkpoint inhibition immunotherapy. It has been clearly demonstrated that the degree of tumour hypoxia correlates with immunosuppressive M2 macrophage infiltration, while it is inversely proportional with intratumoral CD4^+^ and CD8^+^ T cells, the main mediators of anti-tumour immunity (62, 63). Wang and colleagues recently drew attention to macrophage-derived Gal-3 in breast cancer models, showing that M2-polarized macrophages are activated and secrete Gal-3 specifically in hypoxic regions, promoting angiogenesis and vascular mimicry in endothelial monolayers (HUVECs). Induction of Gal-3 was found to be caused by ROS production and NF-κB activation and nucleation. As macrophage depletion was already known to reverse the consequences of hypoxia and abrogate angiogenesis, they were able to reproduce the same result by selectively targeting Gal-3 expression (64). In NSCLC, hypoxia-induced Gal-3 increased localization of RhoA to the plasma membrane, thus activating cell motility (65). Le and colleagues were among the first to report that Gal-1 upregulation is a direct result of hypoxia in HNSCC, being inversely proportional to T-cell infiltration (66). Later, it was further demonstrated that Gal-1 upregulation in hypoxic conditions is HIF-1-dependent and that Gal-1 knockdown significantly reduces hypoxia-induced invasion and migration of colorectal carcinoma cell lines (60).

### Effects of mutated oncogenes

In a very recent publication, Funkhouser and colleagues studied in parallel the expression of 50 known cancer-critical genes in tumor fragments from patients with breast and lung cancer and the profiles of galectins in the serum of these patients. They discovered that gain-of-function mutations in the *KIT* proto-oncogene are consistently associated with elevated serum levels of galectins -1, -3, -8, and -9 in breast cancer and galectin-1 in non-small cell lung cancer patients. *KIT* codes for the receptor tyrosine kinase c-KIT, often upregulated in various cancers, leading to enhanced cell survival and proliferation. The authors suggest that c-KIT may have a role in the increase of galectin abundance in circulation. This could be either through its altered glycosylation pattern in the presence of a mutation, causing increased galectin secretion, or as a result of intracellular mechanisms following its activation by the stem-cell factor (SCF), leading to increased transcription of galectins (67).

### Effects of cytokines and drugs

Galectin-9 expression in tumour cells was found to be induced by extracellular cytokine signaling. More precisely, in growing tumours, DCs and cancer cells secrete IFNβ, while T cells produce IFNγ. Both cytokines were found to upregulate not only Gal-9 expression by cancer cells, but also its secretion. Interestingly, expression but not secretion of Gal-9 required a functional EGFR (68). Jabbari and colleagues, also showed that Gal-9 expression is induced by various types of chemotherapy, which had enhanced cytotoxicity when combined with blockade of Tim-3, the ligand of Gal-9 on the T cell surface (69).

## Role of galectin dysregulations in general cancer biology

### Influence on intranuclear oncogenic processes

Galectins are present in the nucleus, influencing gene expression at the transcriptional and post-transcriptional level. At the former, Gal-3 was found to upregulate oncogenes *Cyclin D1* (70) and *MUC2* (71), by enhancing and stabilizing the binding of their respective transcription factors, CRE and AP-1, to their promoter sites. Direct interaction with the transcription factor TTF-1 also endows cancer cells with superior proliferative capacity (72). Moreover, in the nucleus of breast carcinoma cells, Gal-1 is reported to interact with FoxP3, a transcription factor suppressing potential oncogenes *MYC*, *ERBB2* and *SKP2*, dampening its tumour-suppressive features (73). Another transcription factor, FoxD1, is reported to form a positive regulatory loop with Gal-3, promoting cancer aggressiveness (74). In the context of its perturbed distribution between the nucleus and the cytoplasm, Gal-3 was found to translocate to the nucleus *via* the importin-α/β complex, mediated by the protein’s C-terminal region (75). Of note, phosphorylation of Gal-3 at Ser^6^ has been shown to be crucial for the exertion of its malignant gene regulatory activity and could be a target of therapeutic experimentation (72). Post-transcriptionally, Gal-3 and Gal-1 have been shown to participate in the pre-mRNA splicing machinery, without binding RNA directly, in a carbohydrate-independent manner. Various works demonstrate their interaction with core polypeptides of the spliceosome, inducing significant alterations in the splicing of several cancer-related genes (76–79). By separately inhibiting Gal-1 and Gal-3, it was proven that they bind a common splicing partner, thus being functionally redundant (78) and double inhibition significantly altered the splicing of several genes (77). Interestingly, the amino-terminal domain of Gal-3 was found to inhibit the incorporation of Gal-1 into the spliceosome (78).

### Influence on cellular stress responses

A recent publication focuses on Gal-3 activity in response to stress. Changes in the environment can generate conditions of cell stress, resulting in a multitude of metabolic changes. Forcing stress by thapsigargin administration, this group revealed that Gal-3 localizes in the endoplasmic reticulum (ER)-mitochondria interphase. By downregulating the mRNA half-life of USP14 and SEL1L, Gal-3 prevented the activation of the ER-associated misfolded protein degradation pathway (ERAD) and blocked pre-apoptotic mitochondrial fission (80). These data emphasize on the importance of Gal-3 in preserving ER-mitochondrial integrity and cell survival upon stress. Other works demonstrated a link between Gal-3 and the Bcl2 protein family. Bcl2 is an anti-apoptotic protein, controlled by the master regulator of apoptosis, p53. Harazono and colleagues showed that Gal-3 bears a conserved motif also identified in Bcl2 (NWGR), which allows binding and sequestering the pro-apoptotic protein BAX. This leads to BAX oligomerization, in a similar way to Bcl2 and ultimately to the prevention of apoptosis (55, 81) and resistance to chemotherapy (82) (**Figure 2**).

Another notable finding is the contribution of Gal-1 and Gal-3 to Ras-mediated oncogenic signaling. In many cancers, mutations in the Ras genes endow cells with enhanced growth and resistance to cell death mechanisms. Both galectins were found to interact with Ras proteins, stabilizing them in the cell membrane, but inducing different oncogenic pathways. Gal-1 increased the ERK downstream pathway and Gal-3 the Raf-1 and PI3-K activity (55, 81, 83). Further works demonstrated that Gal-3 is an integral component of the K-Ras-GTP nanocluster in the plasma membrane, determining the magnitude of its activity (84). Disruption of Gal-3 binding to the cell surface receptor integrin αvβ3 was suggested to be a potential therapeutic target in tumours with uncontrollable K-Ras activity (85). Besides, it was demonstrated in neutrophils that intrinsic Gal-3 expression reduces the generation of ROS and that this can be abrogated by inhibition of Gal-3 with modified citrus pectin (86).

### Influence on glucose metabolism

**Figure 3:**
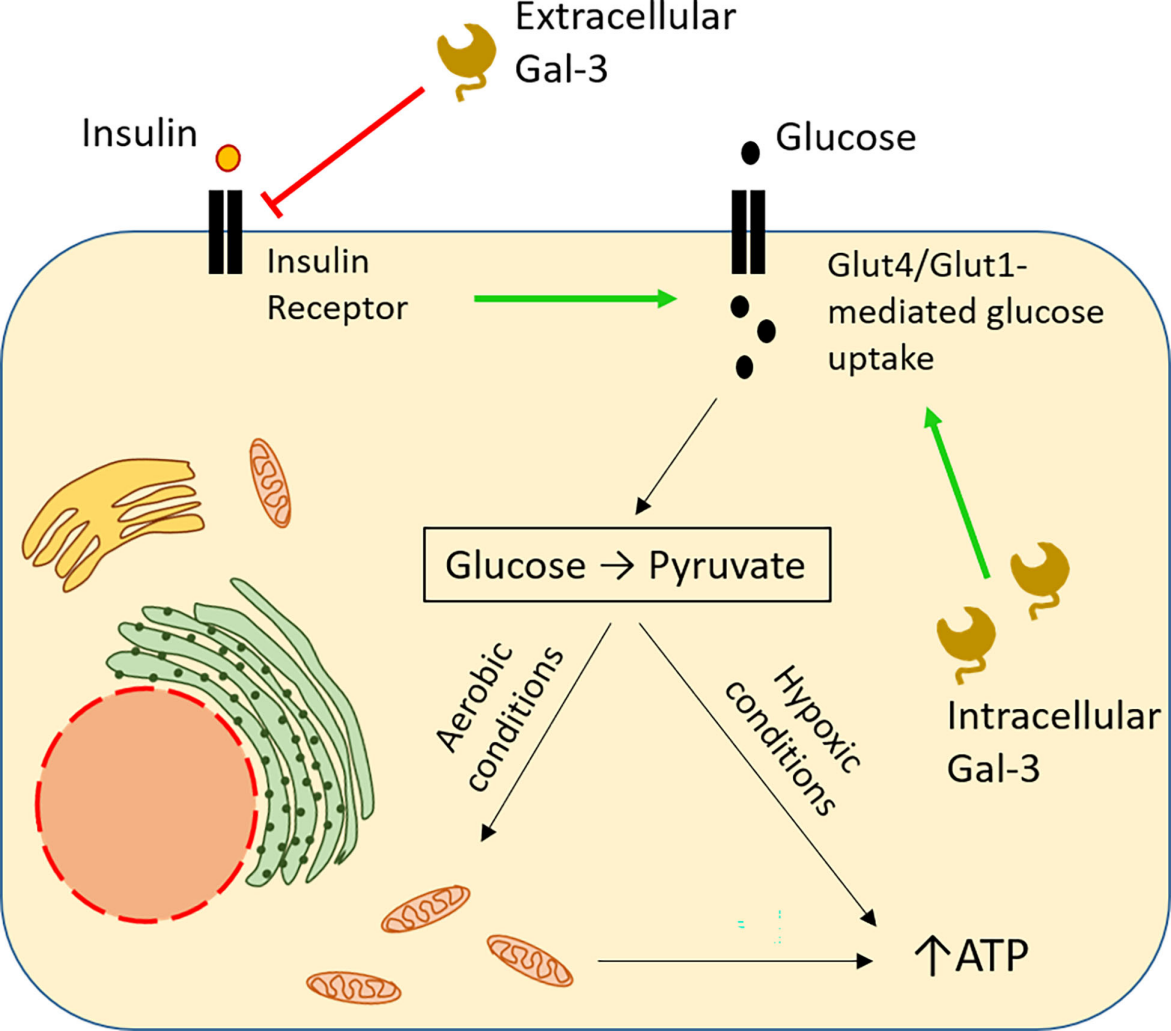
Opposite impact of intracellular and extracellular Gal-3 on glucose uptake. Intracellular Gal-3 mimics insulin binding to its receptor, sustaining expression of the GLUT family of membrane transporters, thus leading to increased glucose uptake by the cell (61, 89 ). In the cytoplasm, glucose is broken down to pyruvate, producing ATP. Under adequate levels of oxygen, pyruvate enters mitochondria, triggering the cell respiration cycle, resulting in more robust ATP synthesis, securing high energy levels for the cell. In contrast, extracellular Gal-3 blocks glucose uptake by the cell, functioning as an antagonist of insulin and preventing binding of the latter to its receptor (91). Hypoxia frequently occurs in the tumour microenvironment forcing the cells to switch to anaerobic glycolysis whose ATP yield is low. Therefore, the needs of external glucose are strongly enhanced in malignant and nearby non-malignant cells. High production of Gal-3 endows the malignant cells with a double selective advantage. Glucose intake is enhanced by internal Gal-3 in malignant cells while it is reduced in non-malignant cells by secreted external Gal-3.

In hypoxic tumour microenvironments, increased glucose uptake is essential for cell survival, as cells switch from oxidative phosphorylation to glycolysis (87) [also elegantly explained in (88)]. Studies in diabetes shed significant light on the implication of intracellular Gal-3 in glucose metabolism. In type 2 diabetes, Gal-3 is upregulated in various tissues and organs. Application of a high-fat diet in Gal-3 KO mice resulted in reduced tissue glucose uptake and greater hyperglycemia, compared to wt mice. This is consistent with a reduction in the expression of the glucose transporter Glut-4 in the absence of Gal-3, with a concomitant reduction in insulin levels (89). In humans, in hypoxic breast and lung carcinomas, Gal-3 has been also shown to be simultaneously upregulated with another glucose transporter, Glut-1 (61). These data suggest that Gal-3 upregulation could potentially sustain a high degree of glucose uptake, even in the absence of insulin signals. Reducing glucose availability by switching from a high-fat to a normal diet downregulates the glucose transporter Glut-4 [discussed in (90)]. However, extracellular Gal-3 has opposite effects on cellular glucose uptake. For example, obesity is characterized by a strong detection of Gal-3 in circulation. Using an obesity-simulating model, Li and colleagues proved that circulating Gal-3 induced systemic insulin resistance. By direct interaction with the insulin receptor (IR), extracellular Gal-3 mediated Glut-4 downregulation and reduced glucose uptake (91). In the context of cancer, this would mean that malignant cells releasing large amounts of Gal-3 could gain access to an increased glucose reservoir by blocking its uptake by adjacent tissues in a paracrine way (**Figure 3**).

### Influence on cell proliferation

In a remarkable discovery, Gal-3 ablation was shown to downregulate the expression of human telomerase reverse transcriptase (hTERT) in gastric cancer cells. Constitutive expression of the normally silent hTERT is a key factor for the immortalization of cancer cells (92), being proportionally correlated with poor patient outcome. La and colleagues observed a link between the mRNA expression levels of hTERT and Gal-3 in gastric cancer cell lines. Knocking out one of them, they discovered that the other one is downregulated. Ablation of hTERT triggers decreased cell proliferation and increased senescence. Gal-3 knockdown or knockout reduced expression of hTERT and led to subsequent senescence which was rescued by forced hTERT overexpression. Immunoprecipitation experiments proved that hTERT activity is regulated by direct interaction with the N-terminal part of Gal-3 (93).

### Influence on cell motility and metastatic processes

The effects of intracellular galectins on cell motility and metastatic processes are complex, not unequivocal and sometimes controversial. In general, the cellular abundance of various galectins strongly affects the cell surface expression of biomolecules, especially glycoproteins and glycolipids with repercussions on cell adhesion properties towards the neighboring cells and the extracellular matrix. However, the consequences in terms of long-range cell motility and metastatic processes are variable depending on the context either *in vitro*, in animal models or in the patients. This complexity is well illustrated by 2 reports dealing with Gal-3 and published the same year, in 2019. Working with the B16 murine melanoma model, Hayashi et al. have either overexpressed Gal-3 in the malignant cells or knocked-out endogenous Gal-3. Both experiments showed that intracellular Gal-3 tends to reduce the metastatic potential of B16 cells, at least in part by negative regulation of the integrin-β3 (94). In contrast, working with the 4T1 murine breast carcinoma model, Pereira et al. showed that Gal-3 tends to increase the metastatic potential of these malignant cells with a concomitant reduction of the surface expression of syndecan-1 and a greater expression of chondroitin sulfate proteoglycans such as versican (95). Apparently with regard to Gal-1, published data are more consistent suggesting that it frequently enhances the metastatic potential of malignant cells. For example, in the 4T1 breast carcinoma model, Gal-1 ablation reduces tumour growth and metastatic spreading. However, the contribution of Gal-1 to the metastatic process in this model seems to be mainly mediated by its immune suppressive effects (96). Using the human gastric carcinoma cell line MGC-803 in athymic mice You et al. showed that Gal-1 promotes the metastatic potential of the malignant cells with a mechanism involving the Sphingosine-1-phosphate receptor 1 (S1PR1). In the same study, the authors reported that the abundance of Gal-1 and S1PR1 detected by IHC on human gastric tumour tissue sections was correlated with a greater frequency of lymph node metastases and a poorer outcome (97). Quite different observations were reported for intracellular Gal-9 in human breast carcinomas. Its high expression in tumour tissue sections is correlated with less metastatic lesions and a better clinical outcome. Simultaneously the authors reported greater cell aggregation and reduced adhesion to the extracellular matrix for MCF7 overexpressing Gal-9 which supposedly was consistent with an anti-metastatic function of intracellular Gal-9 (98).

Extracellular galectins are also suspected to contribute to the metastatic processes especially by their role in the formation of metastatic niches. For example, Gal-3 secreted by malignant cells enhances osteoclast activity in combination with the cytokine RANKL in skeletal metastatic lesions of breast and prostate carcinomas (99). In these pathological contexts, Gal-3 is secreted in a soluble form, either intact (breast carcinoma) or cleaved (prostate carcinoma). Galectins can also be secreted in association with exosomes. For example, this has been reported by our group for Gal-9 and by other groups for Gal-3 (21, 100). To our knowledge the role of tumour exosomes carrying galectins has been reported for immune suppression associated with tumour growth but so far not for the creation of metastatic niches (21, 101).

### Influence on angiogenesis

The impact of galectins on tumour vasculature has been an object of study for more than ten years. Angiogenesis is a well-known pre-requisite for cancer growth (102). Thijssen and colleagues had previously developed an angiostatic peptide, anginex, with demonstrated inhibitory activity on tumour growth, but for a long time were unable to identify its direct target. In 2006, they demonstrated that Gal-1 was the receptor for anginex on the cell surface in various tumour models. Gal-1 exhibited selectively increased patterns of expression in endothelial cells lining tumour vasculature, correlating with staining for proliferation marker Ki67. Binding of anginex to Gal-1 resulted in significant reduction of microvessel density (103). In *in vivo* multiple myeloma models, shRNA-mediated Gal-1 inhibition downregulated pro-angiogenic genes, including MMP9 and CCL2, and upregulated the anti-angiogenic SEMA3A and CXCL10. This led to decreased microvascular density and lower tumour burden (57). Increased Gal-9 expression in endothelial cells and tumour vasculature compared to normal tissues has also been reported (104). Evidence for Gal-3 has also emerged, as dos Santos and colleagues showed that cancer-cell-secreted Gal-3 is bound by endothelial cells, altering the metabolic balance between DLL4 and JAG1, two factors with opposing roles in angiogenesis. More precisely, Gal-3 was found to increase the half-life of the second over the first, leading to a more pronounced VEGFR2 expression and faster tumour growth in mice bearing LLC tumours. On the contrary, this effect was prevented in *LGALS3* ^−/−^ mice (105).

## Impact of galectins on anti-tumour immunity

### Overview of immune tolerance mechanisms related to galectins

Induction of immune tolerance is one major aspect of galectin contributions to the development of human malignancies. This is accomplished through 3 main types of biological processes: 1) alterations of the malignant or stromal cell phenotype distorting their interactions with the immune system; 2) Modifications of the extracellular chemical contexture in the tumour microenvironment; 3) Direct phenotype modifications of the cells of the innate and adaptative immune system. The first type is mainly promoted by intracellular galectins contained in malignant cells while the two others are mainly supported by extracellular galectins.

### Contribution of galectins to defective interactions of malignant or stromal cells with the immune system

Downregulation of the surface expression of MHC-I molecules is one way to impair recognition of target cells by CD8^+^ T-cells. Mathew and Donaldson have shown that endogenous Gal-3 is involved in the internalization of surface MHC-I molecules. Briefly, HeLa cells were treated for 48h with N-acetylglycosamine resulting in enhanced branching of surface glycans and internalization of MHC-I. This last effect was prevented by siRNA knockdown of endogenous Gal-3. Reciprocally, MHC-I internalization was stimulated by extracellular addition of recombinant human Gal-3 (106, 107). In nasopharyngeal carcinoma, a human malignancy involving latent EBV infection, Gal-9 associated with malignant cells distorts their interactions with the immune system in another way. It interacts with STING inside the malignant cells, accelerating its degradation by enhanced ubiquitination. STING degradation then promotes IL-1β and IL-6 secretion by malignant cells which stimulate differentiation of bystander immature myeloid cells into MDSCs which have a strong immunosuppressive potential (108). Galectins can also affect stromal cell interactions with the immune system. Nambiar and colleagues demonstrated that secreted Gal-1 upregulates PD-L1 and Gal-9 expression on endothelial cells. Confirming that this is the result of chronic STAT activation, they proved that Gal-1-mediated impact on PD-L1 and Gal-9 in the tumour endothelium promotes T-cell exclusion, as Gal-1 blockade significantly boosted T-cell infiltration and conferred enhanced response to immunotherapy (109). However, in the context of patients undergoing surgery for hepatocellular carcinomas (HCC), Sideras et al. reported quite distinct observations. Concomitant pre-operative high levels of Gal-9 and PD-L1 in both tumour tissue and circulation, associated with high CD8^+^ TIL counts, predicted very accurately an improved overall and recurrence-free survival in hepatocellular carcinoma (HCC) (110). This could be explained if we take into consideration that both Gal-9 and PD-L1 are upregulated following IFNγ signaling (111, 112), possibly reflecting strong ongoing immunoreactivity, backed by the strong TIL infiltration.

### Modifications of the extracellular chemical contexture in the extracellular environment

Outside the field of cancer, it was found that Gal-1 induction in kidneys exposed to high glucose concentration in type 1 and type 2 diabetes accounts for the development of tissue fibrosis by increasing expression of fibronectin (113). This may have repercussions for cancer biology. Indeed, as explained by Cox and Erler, fibrosis is often involved in cancer development. In tumours, disrupted extracellular matrix (ECM) homeostasis leads to enhanced tumour stroma and desmoplasia (114). Massive collagen deposition in the tumour stroma by activated fibroblasts promotes ECM stiffness, favoring tumour immune escape and progression. Extracellular galectins can also reduce the rise of the local immune response by scavenging inflammatory products, for example end products of advanced glycation (AGEs) or advanced lipidation (ALEs) which result from enhanced glucose metabolism. Binding of such products to their receptors generates an inflammatory response. Extracellular Gal-3 has the opposite effect because it binds AGEs and ALEs, preventing interaction with their receptors, thus contributing to their clearance in an immunosuppressive manner (115).

Some galectins also have the power to directly antagonize cytokine accession to their target cells. Described by Gordon-Alonso and colleagues, secreted Gal-3 accumulates in the tumour extracellular matrix and blocks diffusion of IFNγ by binding its glycans (116). This prevents the creation of a CXCL9/CXCL10/CXCL11 chemokine gradient downstream of an IFNγ signal, which is essential for the recruitment of primed T cells (117). Secreted galectins have been shown to accumulate in the extracellular matrix and the tumour stroma, thus potentially shielding it by blocking the liberation of immune-stimulating signals outside the tumour. Another pivotal work by Blouin and colleagues elegantly supplements the effect of Gal-3 but also of Gal-1 on IFNγ signaling (118). More specifically, unhindered binding of exogenous IFNγ to the IFNγ receptor complex (IFNγR), consisting of the subunits 1 and 2, triggers activation of the JAK/STAT pathway for downstream signaling. Excessive glycosylation of IFNγR2 caused conformational changes in this subunit, eventually impairing JAK activation. Mass spectrometry analysis revealed a 3-fold superior binding of Gal-1 and Gal-3 to the hyper-glycosylated subunit, whereas depletion of the two galectins restored normal signaling. Respectively, the addition of recombinant Gal-3 led to a concentration-dependent decrease of STAT1 tyrosine phosphorylation in IFNγR2 WT fibroblasts. These data imply that the amount of extracellular Gal-1 and Gal-3 bound to IFNγR2 is a key regulator of the subsequent activation of the JAK/STAT signaling pathway by IFNγ. Besides, it is well established that N-glycosylation of human IFNγ is of great importance for its successful secretion and dimerization, protecting it from the action of proteases at the extracellular milieu (119, 120). Generation of stable Gal-9 knockouts of the bladder cancer cell line MB49 led to gradual decline of tumour growth over iterative xenograft transplantations. Replacing cell injections with iterative transplantations of small tumour fragments into new receiver mice, Baloche and colleagues were able to study tumour evolution through time, as the tumour and the immune infiltrate were allowed to “age” together. After 4 passages, Gal-9 WT tumour maintained the same growth rate, while KOs stopped growing. Interestingly, early passage KO tumours had an immune-excluded phenotype, while late passage small tumours displayed robust T cell infiltration and readily produced IFNγ, CXCL9, CXCL10 and CCL5 (121). It is not clear from this work if Gal-9 has a direct interaction with IFNγ, as previously described for Gal-3, but its impact on the chemokine gradient hints towards this assumption. Taken together, these data suggest that Gal-1, Gal-3 and may be Gal-9 thwart cytokine-mediated communications, hampering immune persistence within the tumour and potentially weakening systemic immunity.

### Direct effects on cells of the innate and adaptative immune system

When acting on cells of the immune system, extracellular galectins tend to use diverse and convergent mechanisms to facilitate tumour immune escape. 1) Towards myeloid and non-myeloid cells of the innate immune system, they promote a suppressive phenotype; 2) Towards CD4^+^ and CD8^+^ lymphocytes they tend to strengthen the activity of suppressive cells and to neutralize effector T-cells either by induction of apoptosis, exhaustion or conversion towards a suppressive phenotype. However, like for most regulators of the immune system, the effects of galectins are rarely unequivocal and there are reports of immunostimulatory effects. For example, Gal-9 has been shown to induce cell death in a specific population of T-regs which express Tim-3 and are often present inside tumours, with a strong suppressive activity (68).

Among myeloid cells, DCs (dendritic cells) have a major role in the initiation of the immune response. There are several reports about immunosuppressive effects resulting from functional alterations of DCs by extracellular galectins. For example, extracellular Gal-1 binds CD69 on the surface of DCs, blocking Th17 differentiation and generating immunotolerant DCs (122). In breast carcinomas, binding of extracellular Gal-9 to Tim-3 at the surface of DCs suppresses their production of CXCL9, resulting in a significantly decreased anti-tumour response, especially in the context of a treatment by paclitaxel (123). In older works, extracellular Gal-1 was also found to bind polymorphonuclear (PMN) cells, inhibiting their chemotaxis and endothelial transmigration with possible suppression of these inhibitory activities by addition of lactose (124).

Extracellular galectins also have strong influence on macrophages. Gal-3 promotes the selective polarization of tumour macrophages towards the pro-tumourigenic M2 type and increases the production of the immunosuppressive cytokines IL-4, IL-10 and IL-13 [reviewed in (40)]. In turn, Jia and colleagues demonstrated that M2-derived Gal-3 leads to enhanced recruitment of M2 macrophages, in a positive feedback loop mode (125). Gal-9 has also been identified to promote M2 polarization, potentially through ligation with the Dectin-1 surface receptor and CD206 (34, 126). The plasticity of tumour-associated macrophages between M1 and M2 polarization is a crucial component in the connection between cancer and inflammation [presented in (127)].

Galectins can impair NK cell functions by various mechanisms in diverse types of contexts. In the murine glioma model GL26, Gal-1 released by malignant cells or present on their surface plays a key role in blocking tumour rejection, mainly by inhibitory effects on NK cells (128). Extracellular Gal-3 has been identified as a ligand of NKp30, a NK cell surface receptor normally triggering NK cell activation and IFNγ release (129, 130). Gal-3 binding to NKp30 results in the inhibition of NK-mediated tumor cell lysis *in vitro* and in xenografted malignant cells (129). However, somehow inconsistent data have been published, based on experiments made using the B16 murine melanoma model. Syngenic mice KO-ed for Gal-3 (Gal-3^-/-^) seem to have a better anti-tumour NK response (131). This might result from indirect changes in NK cell physiology, while the inhibitory effect of NKp30 binding is direct. Finally, extracellular Gal-9 has been reported to impair NK cell functions in human and mice, inhibiting both cytotoxicity and cytokine production (132). This is consistent with several publications dealing with the protective effect of Gal-9 released by trophoblastic cells at the foeto-maternal interface. This effect is due to a large extent to NK cell inhibition (133, 134).

MDSCs (myeloid-derived suppressor cells) promote cancer development by various mechanisms, mainly by a suppressive activity on effector T cells. For example, they produce peroxynitrites inactivating CD8^+^ T lymphocytes by nitrosylation of their TCR. They also release TGF-β and IL-10 which favor Treg development. In addition, they have pro-angiogenic activity and can enhance the survival of tumor stem cells (135). Extracellular Gal-1 was reported to enhance the activity of MDSCs. In myeloma, Gal-1 released by malignant cells binds CD304 at the surface of monocyte-derived MDSCs enhancing their expansion. In myeloma patients, there is a correlation between the abundance of circulating M-MDSCs and the plasma concentration of Gal-1 (136). In the murine Lewis Lung Carcinoma model Gal-3 has been reported to enhance the migration of MDSCs in the tumor microenvironment, triggered by cisplatin treatment. This effect seems to be related to the binding of Gal-3 to CD98 at the surface of MDSCs (137). Well before that, Gal-9 was shown to enhance the expansion of granulocytic MDSCs in mice (138). More recently, a study on primary and secondary resistance to anti-PD1 antibodies in lung carcinoma patients has suggested a role for MDSC expansion linked to the activation of the Gal-9/Tim-3 axis. There is also evidence from *in vitro* experiments that Gal-9 can stimulate MDSC differentiation and expansion by mechanisms independent of Tim-3 involving Gal-9 entry in target cells and STING degradation (108).

**Figure 4:**
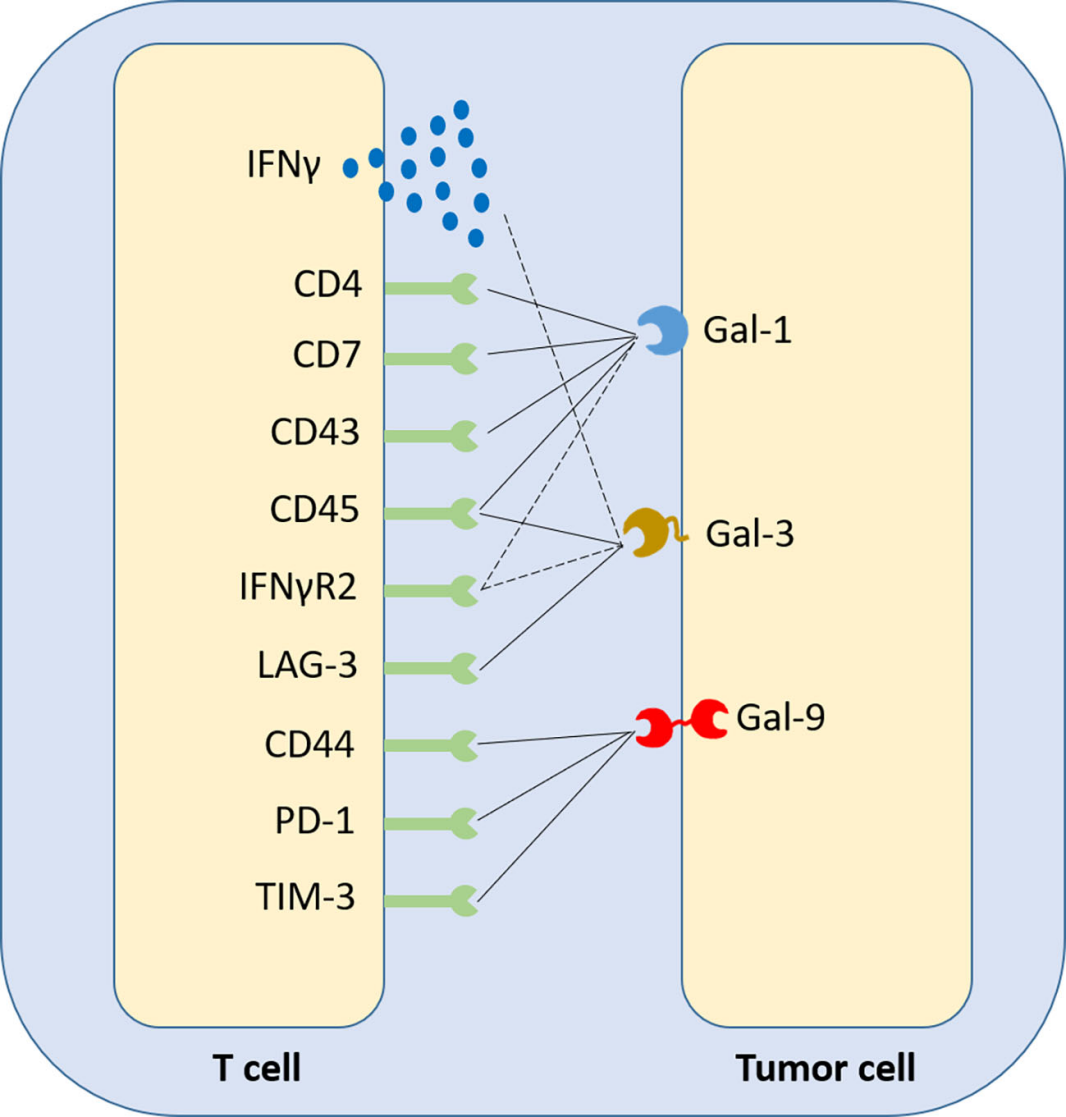
Synthetic representation of the crosstalk between galectins and T-cell surface receptors. Gal-1, Gal-3 and Gal-9 have been identified to be ligands of various membrane receptors, acting as checkpoint inhibitors. Depending on the member of the galectin family and the biology of the respective T-cell membrane receptor, or secreted cytokine, the immunosuppressive effect of galectins on T cells may have three distinct forms; two direct, causing T-cell apoptosis or a switch towards an exhausted phenotype (solid lines) and one indirect, suppressing T-cell-generated extracellular stimuli that could induce a greater immune response if they exited the tumour microenvironment and reached circulation (dashed lines).

Gal-1 expression in the tumour inversely correlates with CD4^+^ and CD8^+^ T cell infiltration in patient samples (9). It was revealed though, that elevated tumor Gal-1 expression in colorectal carcinoma correlates, in both mouse tumours and patients, with a signature of CD8^+^CD122^+^PD-1^+^ Tregs, contributing to an immunosuppressive phenotype and favoring poor prognosis (139). On the T cell membrane, Gal-1 binds mainly CD45, but also CD7, CD43 and CD4, while Gal-3 binds LAG-3 and, secondarily, CD45, while Gal-9 binds Tim-3, CD44 and PD1, promoting apoptosis or exhaustion (68, 140) (summarized in **Figure 4**). The interaction of cancer cell-derived Gal-9 with Tim-3 on Th1 and Th17 cell surface has been identified to favor pro-tumour Th2 responses, as CD4^+^ T cells of this subtype do not express Tim-3. In B cell leukemia, strong expression of PD-L1 and Gal-9 on leukemic B cells correlated with impaired T cells, co-expressing PD1 and Tim-3. PD-1 blockade only yielded modest benefit. In contrast, forced restoration of Akt/mTORC1 signaling in T cells upregulated the glucose transporter Glut1 and glucose uptake and downregulated PD1 and Tim-3 (141). As a matter of fact, unlike naïve subsets, activated T cells switch to glycolysis and heavily rely on glucose uptake. In contrast, activated Tregs continue to rely on oxidative phosphorylation (142). Interestingly, although Gal-1 and Gal-3 bind their respective T-cell ligands with similar affinities, studies suggest that only the latter induces direct apoptosis (143). Gal-1 is suggested to bind T cells and mainly modify their profiles, as it attenuates IFNγ production and triggers IL-10, without altering significantly T-cell viability (144).

In the highly metastatic breast cancer murine model 4T1, silencing of Gal-1 markedly reduced formation of metastases in the lung, associated with reduced Tregs in tumours, lymph nodes and the spleen. Injection of WT 4T1 cells on the opposite flank of the mouse restored the typical metastatic potential, suggesting a systemic modulation of the immune response by Gal-1 (96). Kared et al. have reported in the context of Hepatitis C a balance between Gal-9 released by Tregs and IL-21 released by Th17 cells, with a capacity of IL-21 to protect effector CD4^+^ and CD8^+^ T cells from cell death and/or exhaustion induced by Gal-9. Predominance of the Gal-9 stimulus would favor the transition to a chronic disease (145). Finally, Yang and colleagues recently discovered that Gal-9 binds both Tim-3 and PD-1, but binding of the second prevents the induction of Gal-9/Tim-3-mediated apoptosis (68). It is noteworthy that anti-PD1 currently used in immunotherapy blocks PD-L1 but not Gal-9 binding to PD1 (68). Similarly, anti-Tim-3 currently used in immunotherapy blocks phosphatidylserine and CEACAM1 but not Gal-9 binding to Tim-3 (146).

### Conclusion on immune tolerance mechanisms related to galectins

Overall, the data presented in this chapter clearly show that Gal-1, Gal-3 and Gal-9 are all major players in cancer-related immune suppression. Many of their effects converge towards the transformation of the tumour microenvironment into a non-accessible, immune-excluded compartment. The so-called “cold” tumours with an immune-excluded phenotype consistently present an abnormal expression of galectins (147). This is in line with the fact that separate depletion of each of these galectins in tumour models overexpressing them let to increased T-cell infiltration of the tumour and enhanced antitumour immunity (109, 121, 148–150).

Capalbo and colleagues showed that a high expression of Gal-3 in biopsies of PD-L1^+^ NSCLC tumours prior to treatment with anti-PD1 successfully distinguished the non-responders from the responders (151). This work is an example of galectins’ potential to provide precious information regarding the immune landscape of a diagnosed tumour and selectively assess whether it qualifies for direct immunotherapy or not.

## Galectin targeting and monitoring in cancer therapy

### Small inhibitory molecules (galactoside analogs)

As lactose has been identified as a pan-galectin inhibitor, lactose-containing human milk glycans (HMGs) have been shown to bind human galectins, except for Gal-2. However, one issue that needs to be tackled is that only 1% of HMGs reach circulation, as shown in infants (152). Set aside natural inhibitors, the development of the first synthetic galectin inhibitors has been constantly gaining interest in the last years. The most advanced forms of inhibitors with high affinity include modified large complex carbohydrates (like plant-derived pectins) and small-molecule inhibitors, developed according to the 3D structure of specific galectins (153) (summarized in **Table 2**). In radiotherapy-resistant prostate cancer PCa cells, the treatment with the Gal-3 inhibitor modified citrus pectin (MCP) led to radiosensitization of the cells, increasing ROS production and apoptosis (155). Recently, modified apple pectin (belapectin or GR-MD-02) exhibited significant synergy with anti-OX40 immunotherapy in mice bearing various tumour models. Combined administration of the two treatments prolonged survival, enhancing CD8^+^ T-cell infiltration and reducing monocytic MDSCs and MHC-II^hi^ macrophages (156). Given the size of such molecules however, the ability of modified pectins to target intracellular galectins remains questionable. On the other hand, emerging small-molecule inhibitors have exhibited variable ability to inhibit intracellular galectins, gaining increasing interest (157). Oral administration of GB1107, a new Gal-3 antagonist, along with PD-L1 blockade boosted the CD8^+^ T cell infiltration in lung adenocarcinoma, reversed macrophage polarization towards M1 and increased the levels of produced IFNγ (158). Another oral Gal-3 inhibitor, GB1211 was recently shown to substantially increase the binding of pembrolizumab and atezolizumab, in cancer cell lines overexpressing PD-1 and PD-L1 respectively (159), thus complementing the findings of Capalbo and colleagues (151). In Gal-1-overexpressing hepatocellular carcinoma (HCC), the novel Gal-1 inhibitor OTX008 compensated the downregulated negative regulator of Gal-1 miR-22, blocking cell growth and reducing tumour size, in combination with the angiogenesis inhibitor sorafenib (33). In the same work, it was found that Gal-1 overexpression correlated with bad prognosis and enhanced growth and metastasis by upregulating the Golgi transmembrane protein endoplasmic reticulum 1 (RER1). OTX008 was further found to downregulate proliferation, invasion and angiogenesis in a large variety of cancer cell lines *in vitro*, but also in immunocompromised mice bearing ovarian carcinoma xenografts (160). Mainly, OTX008 was proven to counteract Gal-1-mediated activation of the ERK1/2 and AKT-dependent survival pathways. An inhibitor of Gal-3, GCS-100, proved to have significant synergy with BH3 mimetics (Bcl2 antagonists). Treatment of AML cells with GCS-100 increased the sensitivity to BH3 mimetics, leading to enhanced apoptosis, largely depending on the presence of a WT p53 (48).

**Table 2:** Summary of the types and characteristics of candidate galectin inhibitors that have entered or may enter clinical evaluation in the context of cancer therapy.

Galectin Inhibitor	Target Galectin	Drug type	Recipient medical condition	Clinical Trial ID
PectaSol-C MCP (Modified Citrus Pectin)	Gal-3	Modified large complex carbohydrate	Biochemically relapsed prostate cancer (BRPC)	NCT01681823
GCS-100 (Modified Citrus Pectin)	Gal-3	Modified large complex carbohydrate	Chronic Lymphocytic Leukemia	NCT00514696
GR-MD-02 (Belapectin)	Gal-3	Modified large complex carbohydrate	Melanoma, Non-small Cell Lung Cancer, Squamous Cell Head and Neck Cancer,	NCT02575404, NCT02117362
GB1211	Gal-3	Small molecule	Healthy subjects for evaluation of safety and tolerability, NSCLC	NCT03809052, NCT05240131
GM-CT-01 (DAVANAT)	Gal-3 (and potentially Gal-1 (154),)	Modified large complex carbohydrate	Colorectal Cancer, Prostate Cancer, Lung Cancer, Head and Neck Cancer, Breast Cancer	NCT00054977
OTX008	Gal-1	Small molecule	Solid Tumors	NCT01724320
AP-74 M-545	Gal-1	DNA aptamer	Lung cancer	Currently not under clinical investigation
Lyt200	Gal-9	Monoclonal antibody	Metastatic Solid Tumors (Cholangiocarcinoma, Pancreatic Cancer, Colorectal Cancer)	NCT04666688

### Other inhibitory agents

Other ways to target galectins under investigation include the use of antibodies, aptamers and nanoparticles. The use of a Gal-1-targeting DNA aptamer (AP-74 M-545) prevented the binding of Gal-1 to CD45 and increased CD4^+^ and CD8^+^ T cell influx, suppressing tumour growth in immunocompetent but not in immunocompromised mice (161). In two distinct works, targeted disruption of Gal-1–N-glycan interactions eliminated hypoxia-driven angiogenesis and suppressed tumourigenesis *in vivo*. In addition, these works confirmed the impact of hypoxia on abnormal vascularization, as anti-Gal-1 antibodies gave promising results in increasing the efficacy of anti-VEGF treatment (56, 162). In mice bearing Gal-1-overexpressing glioblastomas, intranasal administration of nanoparticles loaded with Gal-1-targeting siRNA remarkably transformed the TME. Gal-1 blockade through nose-to-brain transport led to reversed macrophage polarization and increased CD4^+^ and CD8^+^ T-cell infiltration, while exhibiting synergy with dendritic cell vaccination and PD-1 blocking (163). Interestingly, Tiraboschi and colleagues found that low dose docetaxel treatment inhibits Gal-3 expression in prostate cancer cells, leading to a reconditioned TME and a superior response to tumour vaccination in a post-surgery mouse model (164). Targeting of Gal-9 is also under investigation, as newly developed Gal-9-neutralizing antibodies have exhibited an ability to prevent Gal-9-mediated T–cell death in mouse tumour models (165). One such antibody, Lyt-200, is currently under clinical investigation in phase I/II trials for its safety and efficacy after encouraging data from treated primates.

## Summary and future perspectives

Galectin upregulation, subcellular localization and activity plays a major part in cancer progression and resistance to therapy through a spectacular variety of functions. So far, galectins are considered not to be oncogenic drivers in human malignancies. However, emerging evidence suggests that they may act early enough, in association with other factors, such as viral latency, to be considered oncogenic (166). Multiple examples presented in this review show that they can be harnessed to multiple aspects of tumour development, both inside the malignant cells and in the tumour microenvironment. One common characteristic of galectins is reducing the accession of immune cells to the tumour area by remodeling the tumour stroma and ECM.

Therefore, they contribute to the pattern of immune-excluded tumours, where lymphocytes accumulate at the tumour periphery, indicating antigen recognition, but fail to penetrate inside the tumour nodules. This is a major reason to develop therapeutic agents for galectin inhibition. Despite all the aforementioned promising features of galectin targeting, its late entry into clinical evaluation might suggest that the scientific community has treated them so far only as supplementary immune checkpoints and not as polyvalent orchestrators of cancer progression, involved in all hallmarks of the disease.

Experimental inhibition of extracellular galectins has yielded some very promising results in combination regimens both in pre-clinical models and early clinical trials (**Table 2**). Therefore, we believe that profiling tumour and circulating galectins will become more and more important in order to accurately assess the immunological status of tumour-bearing patients. In the medium term, galectin targeting might become a powerful adjuvant of various oncological therapeutic tools like chemotherapy, immune checkpoint inhibition, CAR-T-cell infusion or anti-tumour vaccination.

## Author contributions

All authors listed have made a substantial, direct, and intellectual contribution to the work and approved it for publication.
